# Association of antegonial notch size with craniofacial morphology and masticatory muscle dimensions

**DOI:** 10.1038/s41598-025-08800-x

**Published:** 2025-07-02

**Authors:** Tatiana Sella Tunis, Dana Rachmiel, Yoav Shapinko, Evgeny Weinberg, Waseem Abboud, Israel Hershkovitz

**Affiliations:** 1 Department of Orthodontics, Maurice and Gabriela Goldschleger School of Dental Medicine, Tel Aviv University, 69978 Tel Aviv, Israel; 2 Department of Anatomy and Anthropology, Gray Medical School, Gray Faculty of Medical and Health Sciences, Tel Aviv University, 69978 Tel Aviv, Israel; 3 Department of Periodontology and Oral Implantology, Maurice and Gabriela Goldschleger School of Dental Medicine, Tel Aviv University, 69978 Tel Aviv, Israel; 4 Department of Oral Biology, Maurice and Gabriela Goldschleger School of Dental Medicine, Tel Aviv University, 69978 Tel Aviv, Israel; 5 Department Oral and Maxillofacial Surgery, Maurice and Gabriela Goldschleger School of Dental Medicine, Tel Aviv University, 69978 Tel Aviv, Israel; 6 The Dan David Center for Human Evolution and Biohistory Research and The Shmunis Family Anthropology Institute, Tel Aviv University, 69978 Tel Aviv, Israel

**Keywords:** Antegonial notch, Facial growth, Facial types, Mandibular morphology, Masticatory muscles, Sex, Anatomy, Medical research

## Abstract

The antegonial notch (AGN) is a structure that has been variably described across studies, with diverse findings regarding its role and association with craniofacial morphology, which is attributed to differing methodologies and the lack of control for sex and size. This study aimed to elucidate the variability of the AGN and examine its association with facial types, skeletal and muscular parameters. This study was carried out using the CT scans of 311 individuals aged 18–95 years. Facial type, skeletal and muscular measurements were recorded. Mann–Whitney and Kruskal–Wallis tests were conducted to examine AGN area differences between the sexes and facial types. Spearman correlation was used to explore the relationships between AGN and the skeletal and muscular parameters. AGN was found to be sex dependent: it was absent in 22.7% of males and 35.1% of females; its size was larger in males than in females (55 mm^2^ vs. 31 mm^2^) (*p* < 0.001). The AGN area did not vary between the facial types (*p* > 0.073). Its size showed weak correlations with skeletal parameters and no associations with masticatory muscles (*p* > 0.375). Our findings indicate that the AGN is more prevalent and pronounced in males, and that there was no evidence of a functional relationship.

**Supplementary Information:**

The online version contains supplementary material available at 10.1038/s41598-025-08800-x.

## Introduction

The antegonial notch (AGN) is an anatomical structure of the human mandible; it has been assigned numerous terms in the scientific literature, such as “gonial notch”, “pregonial notch”, “pre-angular notch”, “premasseteric notch”, “notch for the facial vessels”, “facial notch”, “facial artery notch”, and “groove for the facial artery”^1,2^. Despite ongoing uncertainty about its development and function, the wide range of terminology used for AGN reflects its broad relevance across dentistry, medicine, and anthropology.

Previous studies have related the size of the AGN to the mandibular vector of growth and its growth potential^3,4^. They suggested that increased notch size was associated with diminished mandibular growth and development. These studies showed that “deeper” AGN cases were associated with a vertical growth pattern, whereas “shallow” AGN cases tended to present with a horizontal direction of mandibular growth^3–5^. Conversely, Kołodziej et al.^6^ concluded, based on their longitudinal sample of untreated patients, that the AGN morphology is not a valid indicator of mandibular growth, since the association between the notch depth and mandibular growth is very weak.

Notch size has also been linked to specific morphological traits. For instance, the “deep” AGN cases were found to have significantly shorter mandibular bodies, shorter ramus heights, retruded mandibles, shallower curves of Spee, greater gonial angles, and increased total and lower facial heights^3,4,7^. Nevertheless, Nakajima and Osato^8^ found a significantly greater AGN in the low gonial angle group compared with the high gonial angle group, which contradicts previous studies. Tomer and Kishani^9^ also found no significant association between the AGN depth and the vertical craniofacial morphology. Based on their findings, the authors suggested that increased masseter activity is associated with deeper AGN. A recent study by Manabe et al.^10^ hypothesized that the notch is formed by the contribution of masticatory muscles that counteract the clockwise growth of the mandible. However, the authors found only weak correlations with two parameters indicating the growth direction (the y-axis and ramus inclination); moreover, the masticatory muscles were not evaluated at all.

Another important point of disagreement is the differences in AGN size between the sexes, which have been noted in previous studies. Kaczkowski et al.^11^ found, based on dried mandibles, sex differences in the AGN depth and anterior length, with significantly longer and deeper notches found in males than in females. Porwolik et al.^12^ also noted AGN size differences between the sexes. A recent study that evaluated the CT DICOM images of adult individuals with normal occlusion concluded that the AGN size and shape can be utilized as an auxiliary characteristic for sex determination in anthropological and forensic disciplines^13^. Nevertheless, Mangla et al.^5^ did not observe sexual dimorphism regarding AGN depth in their sample.

Unfortunately, the literature reports inconsistent findings regarding the size of AGN in the population, the difference between sexes, and the association with other craniofacial parameters. Several factors may partly explain this inconsistency:

1. Utilization of varied methodological tools for AGN size assessment: caliper usage on dry mandibles; panoramic radiographs, which may contain magnification and head postural errors; lateral cephalograms, which could encounter methodological difficulties due to the superimposition of both sides; CT DICOM images, which may encounter difficulties in providing a proper plane of section through the notch.
2. Varied exclusion/inclusion criteria for cases with and without AGN: only a few studies reported the number of individuals with no AGN; other studies excluded such cases or did not report them.
3. Sex control: Most of the studies provided conclusions based on an analysis of a combined sample (males and females); only a few analyzed males and females separately.
4. Size control: The studies did not correct for the general size of the mandible when analyzing the different parameters involved in its expression. The AGN size could potentially be related to the size of the mandible (allometry).

Assessing AGN prevalence and size variation between sexes, along with its associations with craniofacial, mandibular, and muscular parameters, may provide valuable insights into its potential role within the craniofacial complex and masticatory system. If consistent anatomical and functional relationships are identified, the AGN could serve as a clinically relevant landmark across multiple disciplines. For surgeons and anatomists, a better understanding of the AGN morphology and variability is essential for improving surgical precision and anatomical orientation. In forensic contexts, recognizing sex-related differences and morphological patterns may enhance identification accuracy. Additionally, the AGN may serve as a potential marker for evaluating craniofacial growth, thereby having meaningful diagnostic value in orthodontic assessment and treatment planning.

The objectives of this study were to (1) determine the prevalence and size of the AGN in the adult population using CT scans, and (2) examine its association with facial types, mandibular and craniofacial morphology, and masticatory muscle size, while controlling for sex and general mandibular size.

## Materials and methods

### Study sample

The study analyzed 311 head and neck CT scans (n = 163 males; n = 148 females) from a randomly selected adult population. Sample size was calculated using WinPepi software (Compare2, version 3.85), indicating that a minimum of 105 individuals per group (males and females; total n = 210) was required to detect a significant difference in AGN area, assuming an effect size of at least 0.5 SD, a significance level of *p* < 0.05, and a power of 80%.

Inclusion criteria were: teeth at the centric occlusion (maximum intercuspation), intact anterior dentition, and at least two posterior teeth (premolars and/or molars) on each side.

Exclusion criteria included: (1) dental implants or metal restorations interfering with measurements; (2) evidence of orthodontic treatment (e.g., brackets, appliances, or lingual fixed retainers); (3) prior head and neck surgery (as indicated in medical records or visible skeletal signs); (4) prominent facial or mandibular asymmetry; (5) craniofacial, temporomandibular joint, or muscle disorders; (6) trauma; and (7) technically aberrant CT scans.

All CT scans were originally acquired for diagnostic purposes unrelated to this study at Carmel Medical Center, Haifa, Israel (Brilliance 64, Philips Medical Systems, Cleveland, Ohio: slice thickness 0.9–3.0 mm; pixel spacing 0.3–0.5 mm; 120 kV; 250–500 mA; 150–950 slices; matrix size 512 × 512).

This retrospective study was approved by the Ethics Committee of Carmel Medical Center (CMC 11-0066). All methods were carried out in accordance with their guidelines and regulations. The requirement for individual informed consent was waived by the Ethics Committee of Carmel Medical Center, as the CT scans were obtained previously for clinical purposes unrelated to this study.

### Measurement technique

All measurements were taken from the CT scans using a multi-planar reformatting technique in the Extended Brilliance Workspace portal (version 2.6.0.27; Philips Medical Systems, Cleveland, OH, USA; https://www.philips.co.uk/). All unilateral measurements were carried out on the right side. The AGN area and muscles’ cross-sectional area (CSA) were extracted from the CT software using manual tracing of their anatomical borders.

To avoid associations attributable to mandibular size, all measurements were corrected for the general size of the mandible using the mandibular geometric mean (MGM) that was calculated based on the mandibular body height and length, ramus length, symphysis thickness, and inter-gonial distance. All linear measurements were divided by MGM; for area measurements the square root was calculated and then divided by the MGM^14^.

### Measuring AGN

**Fig. 1:**
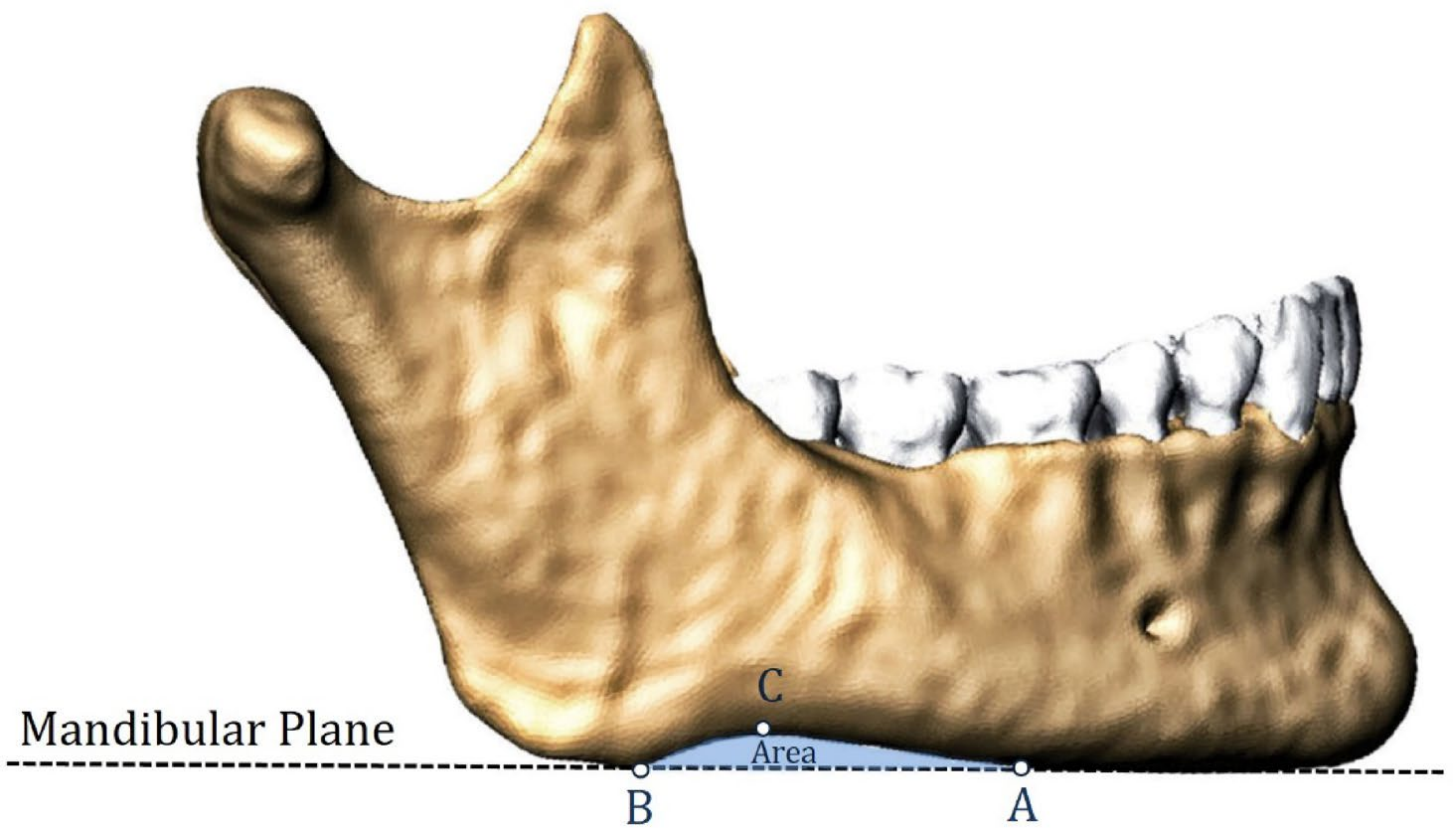
Measurement method for the AGN area (the light blue area). The notch is bordered between the lower rim of the mandibular body and the mandibular plane. Anatomical landmarks defining the notch area: (**A**) the anterior limit of the notch, (**B**) the posterior limit of the notch, and (**C**) the peak of the notch. The volume rendering technique was utilized to create the shape of the mandible.

The AGN area was defined as the region bordered by the lower margin of the mandible and the mandibular plane (MP) following Downs^15^. It was delineated using three anatomical landmarks (Fig. 1), as described by Kaczkowski et al.^11^:

- *Point A*—the anterior limit of the notch, where the inferior margin of the mandible departs from the MP;
- *Point B*—the posterior limit of the notch, where the inferior margin of the mandible meets the MP;
- *Point C*—the peak of the notch, defined as the most distant point on the inferior margin of the mandible from the MP.

AGN was classified into two groups: “absent” and “present” based on its area size. To account for minimal variations due to image resolution and measurement error, AGN was classified as “absent” when the measured area was less than 2 mm^2^, and “present” when ≥ 2 mm^2^. This threshold was introduced to improve the reliability and reduce the misclassification of very small AGN areas.

### Measuring the skeletal components

Mandibular metric characteristics were assessed using 18 different linear, angular, and cross-sectional measurements^14–17^. In addition, six linear and angular parameters were used to evaluate craniofacial size (see Supplementary Tables S1 and S2 online, and Supplementary Fig. S3 online).

### Measuring the muscles of mastication

Three masticatory muscles (temporalis, masseter, and medial pterygoid) were assessed for size following the method described by Weijs and Hillen^18^. The CSAs of the masseter and medial pterygoid muscles were measured by tracing their borders on a plane oriented 30° relative to the FH plane and positioned 3 cm ventrocranially to the gonial angle. The temporalis muscle was traced on a plane parallel to the FH plane and located 1 cm cranial to the zygomatic arch (see Supplementary Fig. S4).

### Facial types

Individuals were classified into three categories: short facial type (SFT), average facial type (AFT), and long facial type (LFT) based on a combination of the following measurements: (1) MP angle, (2) lower anterior facial height, and (3) facial height index, as described by Sella Tunis et al.^19^.

### Statistical analysis

Data were recorded and analyzed using the SPSS software package (IBM Corp. 2023. IBM SPSS Statistics for Windows, Version 29.0.2.0, Armonk, NY: IBM Corp). The Kolmogorov–Smirnov test was used to assess the normality of distribution. Differences in age between sexes were evaluated using the Mann–Whitney test. The Kruskal–Wallis test was employed to detect age differences across facial types. The Chi-square test assessed associations between sex and AGN presence. The Mann–Whitney test was used to compare AGN area (absolute and size-corrected) between sexes. The Spearman correlation was used to find an association between age and the AGN area. The Kruskal–Wallis test was run to find significant differences in the AGN area between the facial types. The Spearman correlation analysis was used to find an association between AGN and the skeletal and muscular parameters. The correlation coefficients were compared between sexes using the Fisher transformation. Statistical significance was set at *p* < 0.05.

### Reliability

The ability to accurately replicate the measurements was calculated using the ICC test following two blinded measurements on 15 different CT scans by a single researcher (T.S.T.), with a 2-week interval between each measurement. An additional independent researcher (I.H.) also conducted the measurements.

## Results

### Reliability analysis

All measurements were found to be reliable. The intratester ICC ranged from 0.838 to 0.995 (*p* < 0.002), and the intertester ICC ranged from 0.706 to 0.989 (*p* < 0.006).

### Demographic features of the sample

The study sample included 163 males (52.4%) and 148 females (47.6%), aged 18–95 years. The mean age was 47.5 ± 19.5 years for males and 50.8 ± 21.1 years for females, with no difference between the sexes (*p* = 0.190). Most of the sample (69.5%, n = 216) consisted of individuals with an average facial type (AFT) (n = 115 males and n = 101 females); the remaining participants were similarly distributed between a long facial type (LFT) (15.8%, n = 49) and a short facial type (SFT) (14.8%, n = 46). The LFT group consisted of 18 males and 31 females, whereas the SFT group included 30 males and 16 females. No difference was found in the mean age between the three facial types (*p* = 0.081).

### AGN prevalence and area size by sex

**Fig. 2:**
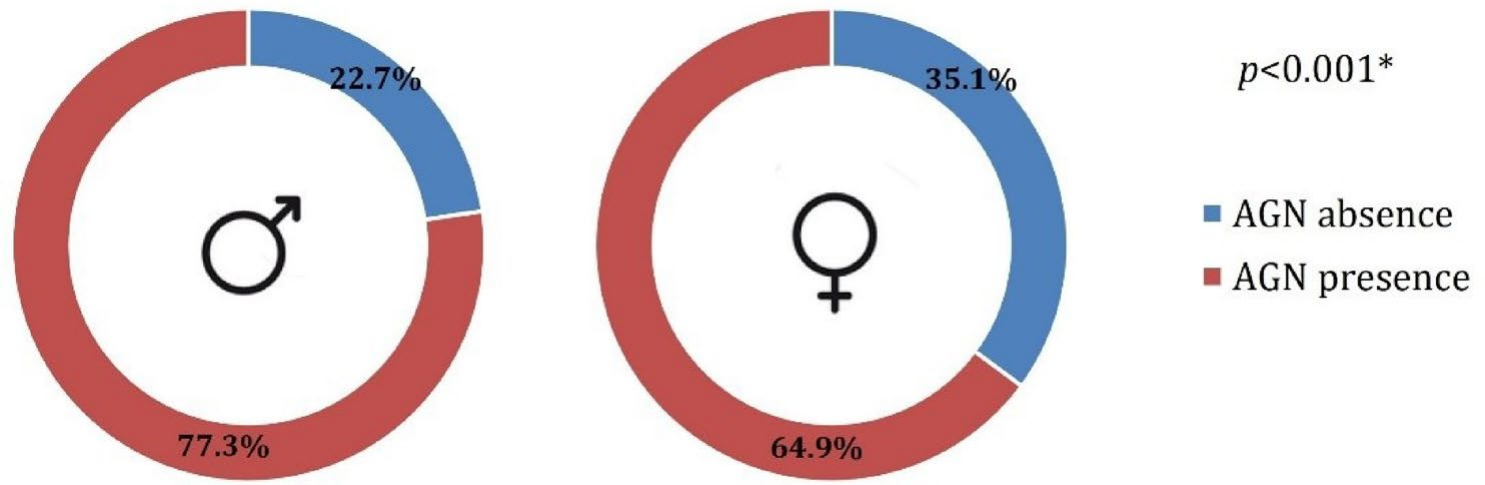
Prevalence of AGN in males and females.

AGN was absent in 22.7% (n = 37) of the males, compared with 35.1% (n = 52) of the females. This difference between the sexes was statistically significant (Fig. 2) (*p* < 0.001).

**Fig. 3:**
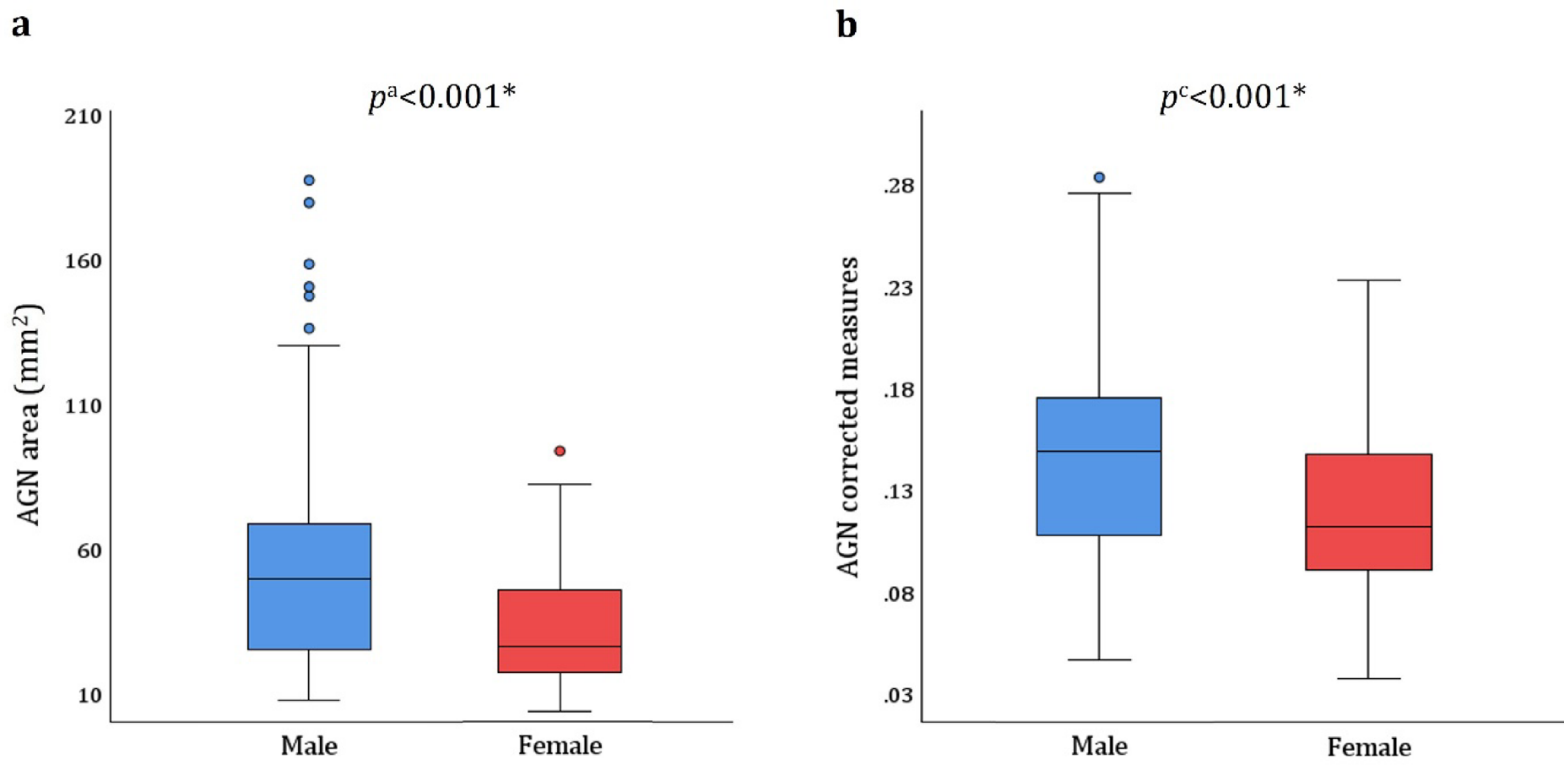
The AGN area in males and females with AGN (area ≥ 2 mm^2^). (**a**) Absolute measures, (**b**) corrected measures. *p*^a^ represents the* p*-value for absolute area size; *p*^c^ represents the *p* value for the corrected area size.

In males with AGN, the mean AGN area (absolute measures) was 54.8 ± 36.5 mm^2^, with a median of 49.2 mm^2^, ranging from 6.9 to 187.3 mm^2^. In females with AGN, the mean AGN area (absolute measures) was 31.1 ± 25.4 mm^2^, with a median of 25.4 mm^2^, ranging from 3 to 93.3 mm^2^. The difference between the sexes was found to be statistically significant for both the absolute and corrected measures (*p* < 0.001) (Fig. 3).

### AGN by age

No correlation was found between age and the AGN area size in either sex. In males, the correlation coefficients were r = − 0.07 (*p* = 0.401) for absolute measures, and r = − 0.08 (*p* = 0.334) for corrected measures. In females, the correlation coefficients were r = − 0.15 (*p* = 0.067) for absolute measures, and r = − 0.16 (*p* = 0.057) for corrected measures.

### AGN prevalence and area size by sex and facial type

**Fig. 4:**
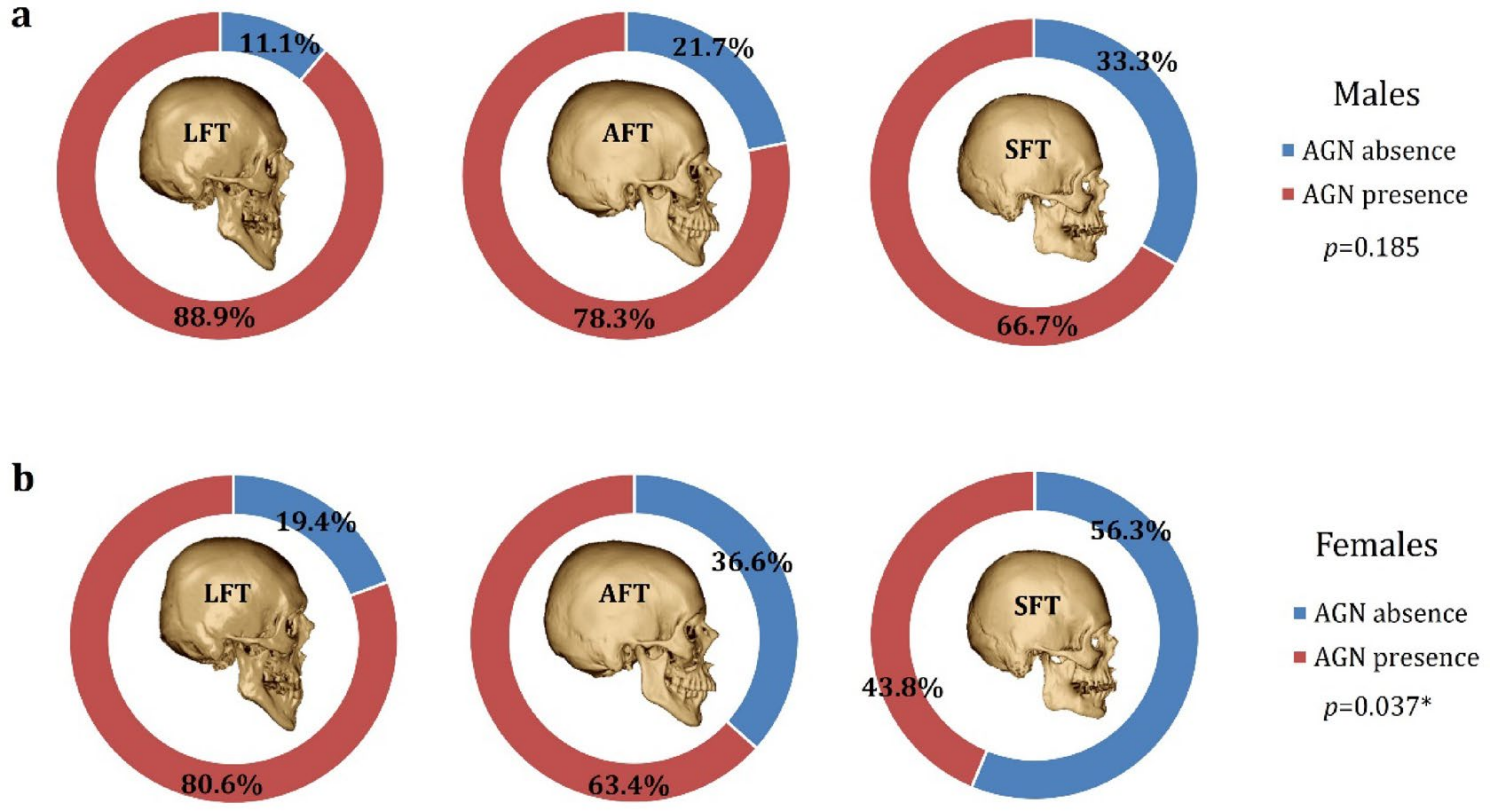
The prevalence of AGN by facial type. *LFT* long facial type, *AFT* average facial type, *SFT* short facial type.

LFT individuals exhibited the lowest frequency of mandibles with an absent AGN, whereas SFT individuals exhibited the highest rate in both males and females. However, this difference was statistically significant only for females (*p* = 0.037) (Fig. 4).

**Fig. 5:**
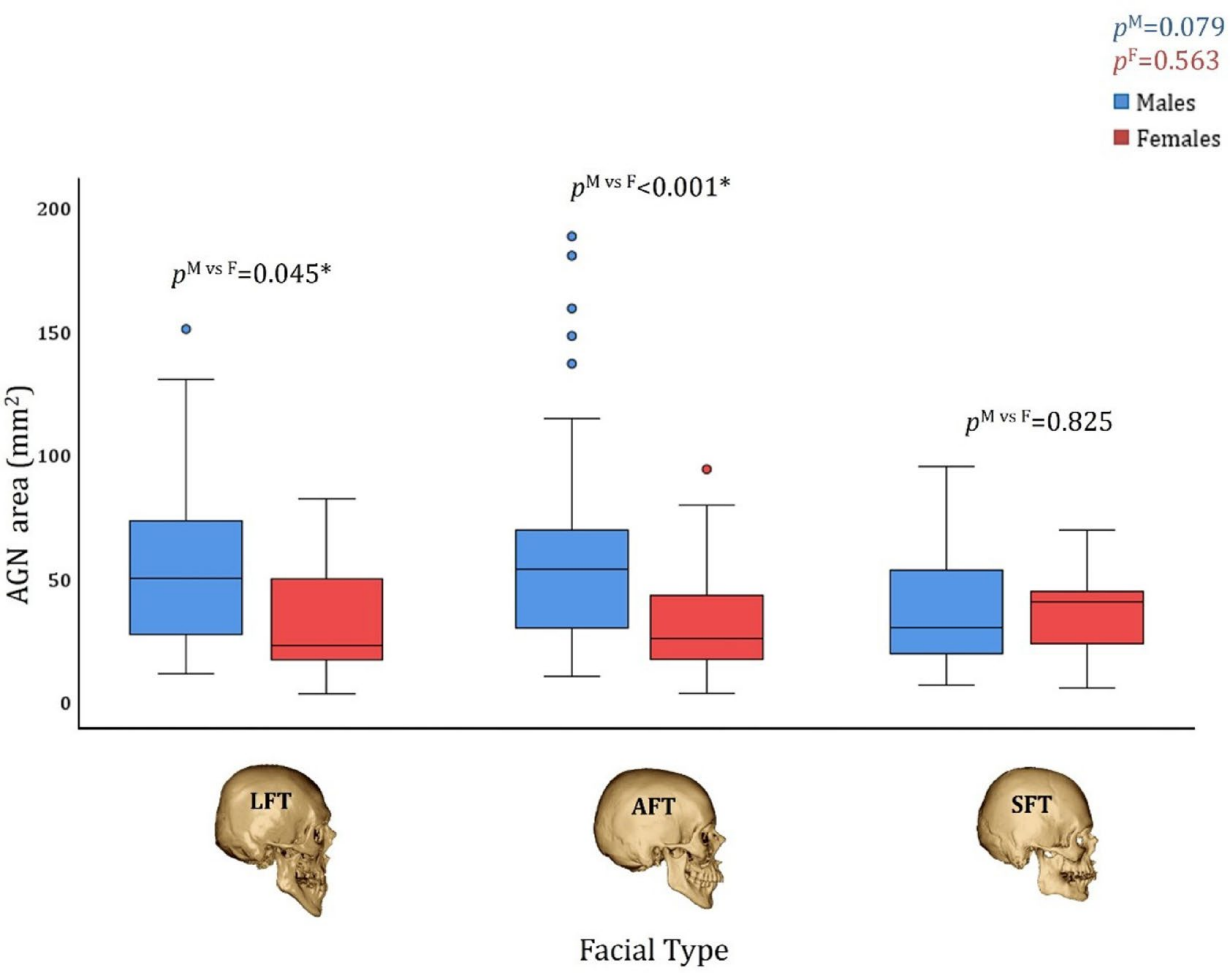
The AGN area size (absolute measures) by facial type and sex in individuals with AGN. *p*^M^ represents the statistical difference between facial types in males; *p*^F^ represents the statistical difference between facial types in females, and *p*^M vs F^ represents the statistical difference between the sexes in each facial type. LFT: Long Facial Type, AFT: Average Facial Type, SFT: Short Facial Type.

**Fig. 6:**
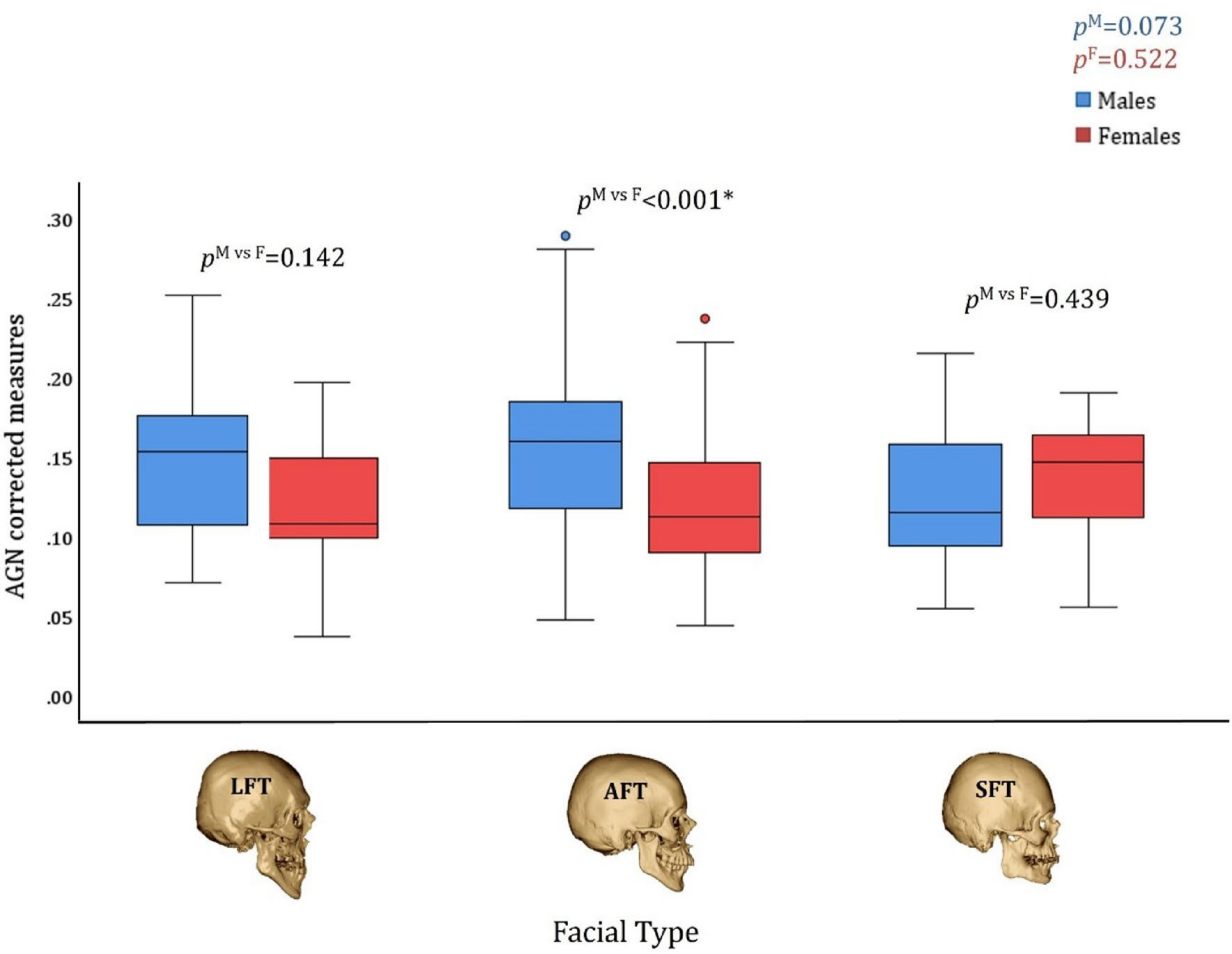
The AGN area size (corrected measures) by facial type and sex in individuals with AGN. *p*^M^ represents the statistical difference between facial types in males; *p*^F^ represents the statistical difference between facial types in females, and *p*^M vs F^ represents the statistical difference between the sexes in each facial type. LFT: Long Facial Type, AFT: Average Facial Type, SFT: Short Facial Type.

In males and females with AGN, the magnitude of the AGN area (both absolute and corrected measures) did not differ between facial types (*p* > 0.073) (Figs. 5 and 6). An interesting finding emerged when comparing the AGN area between males and females with different facial types. Although the AGN area was larger in males than in females, this difference became statistically significant only in AFT individuals after correcting for mandibular size (*p* < 0.001) (Figs. 5 and 6).

### AGN area and the skeletal components

Correlations between AGN area and mandibular skeletal parameters were generally weak (Table 1)^20^. The strongest positive correlations were observed between the corrected AGN area and the gonial angle width (r = 0.36 in males, r = 0.23 in females, *p* < 0.004), with no difference between the sexes (*p* = 0.214). The strongest negative correlations were found between the absolute AGN area and the condylar width (r = − 0.25 in males, r = − 0.31 in females, *p* < 0.001), also with no sex difference (*p* = 0.570). In absolute measures, the AGN area was negatively correlated with all chin parameters, but no correlations were found with symphysis parameters in either sex (*p* > 0.103) (Table 1). Additionally, no significant correlations were found regarding ramus length, mandibular body length, gonial angle, and width measures (the inter-gonial, inter-condylar distance) (*p* > 0.064).

**Table 1:** Correlation coefficients between the AGN area and other mandibular parameters in males and females.

Measurement	Sex	Absolute measures			Corrected measures		
		r	*p* value	*p* ^M vs F^	r	*p* value	*p* ^M vs F^
Chin Thickness	Male	**− 0.24**	**0.002**	0.927	**− 0.23**	**0.004**	0.855
	Female	**− 0.23**	**0.004**		**− 0.21**	**0.011**	
Chin Area	Male	− 0.13	0.086	0.473	− 0.12	0.122	0.656
	Female	**− 0.21**	**0.010**		**− 0.17**	**0.034**	
Chin Width	Male	**− 0.19**	**0.014**	0.355	− 0.15	0.053	0.528
	Female	**− 0.29**	**< 0.001**		**− 0.22**	**0.007**	
Symphysis Thickness	Male	− 0.03	0.742	0.380	− 0.06	0.476	0.598
	Female	− 0.13	0.103		− 0.12	0.164	
Symphysis Height	Male	0.12	0.112	0.792	**0.19**	**0.016**	0.583
	Female	0.09	0.268		**0.25**	**0.002**	
Symphysis Area	Male	0.08	0.335	0.432	0.14	0.077	0.859
	Female	− 0.01	0.868		0.12	0.138	
Ramus Width	Male	0.14	0.066	0.481	**0.21**	**0.008**	0.786
	Female	0.06	0.462		**0.18**	**0.028**	
Ramus Length	Male	0.06	0.435	0.257	0.10	0.188	0.726
	Female	− 0.07	0.428		0.06	0.434	
Mandibular Body Length	Male	− 0.02	0.761	0.600	0.04	0.586	0.930
	Female	− 0.08	0.359		0.05	0.507	
Mandibular Body Height Ratio	Male	**0.19**	**0.028**	0.785	**0.17**	**0.044**	0.586
	Female	**0.22**	**0.016**		**0.23**	**0.015**	
Mandibular Plane Angle	Male	0.15	0.050	0.469	**0.17**	**0.033**	0.524
	Female	**0.23**	**0.006**		**0.24**	**0.004**	
Gonial Width	Male	**0.28**	**< 0.001**	0.123	**0.36**	**< 0.001**	0.214
	Female	0.11	0.173		**0.23**	**0.004**	
Inter-gonial distance	Male	**− 0.17**	**0.032**	0.080	− 0.09	0.253	0.055
	Female	0.03	0.704		0.13	0.129	
Gonial Angle	Male	− 0.15	0.064	0.161	− 0.11	0.174	0.336
	Female	0.01	0.949		0.00	0.984	
Coronoid Process Height	Male	− 0.04	0.591	0.727	0.01	0.855	0.727
	Female	0.00	0.970		0.05	0.583	
Coronoid Process Width	Male	0.14	0.075	0.590	**0.19**	**0.013**	0.308
	Female	**0.20**	**0.017**		**0.30**	**< 0.001**	
Condylar Width	Male	**− 0.25**	**0.001**	0.570	**− 0.20**	**0.010**	0.928
	Female	**− 0.31**	**< 0.001**		**− 0.19**	**0.019**	
Inter-condylar distance	Male	− 0.02	0.770	0.381	0.08	0.285	0.861
	Female	− 0.12	0.146		0.06	0.434	

*p*^M vs F^ represents statistical differences in the correlation analysis between sexes.

*Statistically significant results (*p* < 0.05) are denoted in bold.

The associations between the AGN area and the craniofacial parameters showed only weak correlations (Table 2)^20^. The facial angle was negatively correlated with the AGN area in both sexes (r = − 0.2, *p* < 0.021).

**Table 2:** Correlation coefficients between the AGN area and the craniofacial parameters in males and females.

Measurement	Sex	Absolute measures			Corrected measures		
		r	*p* value	*p* ^M vs F^	r	*p* value	*p* ^M vs F^
Cranial Length	Male	0.13	0.109	0.583	**0.21**	**0.009**	0.927
	Female	0.06	0.490		**0.22**	**0.009**	
Cranial Breadth	Male	− 0.01	0.899	0.794	0.11	0.158	0.594
	Female	0.02	0.768		**0.17**	**0.039**	
Cranial Base Angle	Male	− 0.02	0.840	0.134	− 0.01	0.915	0.135
	Female	**− 0.19**	**0.02**		**− 0.18**	**0.029**	
Facial Breadth	Male	0.02	0.774	0.727	0.13	0.108	0.655
	Female	0.06	0.449		**0.18**	**0.030**	
Facial Height	Male	**0.17**	**0.028**	0.928	**0.26**	**0.001**	0.777
	Female	**0.18**	**0.030**		**0.29**	**< 0.001**	
Facial angle	Male	**− 0.26**	**0.001**	0.518	**− 0.27**	**0.001**	0.777
	Female	**− 0.19**	**0.021**		**− 0.20**	**0.013**	

*p*^M vs F^ represents statistical differences in the correlation analysis between sexes.

*Statistically significant results (*p* < 0.05) are denoted in bold.

### AGN area and the muscular component

No statistically significant correlations were found between the AGN area and the masticatory muscle area, both in absolute and corrected measures (*p* > 0.138) (see Supplementary Table S5 online).

## Discussion

The present study provides data on the AGN prevalence and size derived from CT scans of a normal adult population, aimed at exploring the variability and potential functional significance of this anatomical feature.

To the best of our knowledge, this is the first study to report the prevalence of AGN. Although many previous studies have focused on “deep” and “shallow” AGN, only a few have addressed cases where the AGN is absent^11,13^. Our findings indicate that approximately 23% of males and 35% of females lack an AGN, with a statistical difference between the sexes. In contrast, Maczka et al.^13^ reported an absence of AGN in only 3.6% of their sample, whereas Kaczkowski et al.^13^ observed an AGN presence in nearly 90% of their participants. The discrepancies between the studies are likely attributable to differences in methodology and sample selection^13^.

These findings have relevant anatomical and clinical implications. The AGN is frequently referred to as the “facial notch” due to its close anatomical relationship with adjacent facial neurovascular structures^1^. Specifically, the facial artery, a branch of the external carotid artery, traverses this region as it ascends from the neck to the face. Additionally, the marginal mandibular branch of the facial nerve has been found to lie within 1 cm of the AGN border^21,22^. Owing to this proximity, the AGN has been proposed as a reliable landmark for identifying a “safe zone” during various surgical procedures involving the lower face and mandible^21,22^. As observed in our study, the absence of the AGN represents a relatively common anatomical variation of the mandible, with nearly one-third of individuals lacking this structure. This is clinically significant, as mandibles lacking an AGN may show deviations in the expected course of facial vessels and nerves. In these cases, the use of alternative anatomical reference points should also be considered to ensure safe and accurate surgical access. Preoperative assessment of AGN variability is advisable for accurate surgical planning, and future anatomical studies that will investigate the trajectory of facial neurovascular structures in cases of absent AGN are of particular importance.

Additionally, we found that males exhibit more than twice the AGN area size compared with females. This finding is consistent with previous studies^23–26^ that reported sex differences, with males displaying deeper notches than females. Furthermore, Schutz et al.^24^ observed that the deepest notches were exclusively found in males. These authors suggested that this difference may be related to the greater masticatory mass in males^23,24^ or, alternatively, to the larger mandibular size in males, which could account for the larger AGN. In the present study, we investigated both (1) the impact of the overall mandibular size and (2) the relationship between AGN and the size of masticatory muscles. Our findings confirm that, even after controlling for mandibular size, males still exhibit significantly larger AGN areas than do females, indicating that sexual dimorphism in this structure is independent of the mandibular size.

Regarding the association with masticatory muscles, no correlations were found in either the absolute or corrected measures for both sexes, suggesting that the AGN is not linked to masticatory muscles. Since its location is anterior to the gonial angle and the insertion sites of the masseter and medial pterygoid muscles, the AGN depth was suggested to be associated with masticatory muscle activity and related to the bone apposition at the muscles’ attachment site^10,24^. Unal Erzurumlu et al.^27^ even compared the AGN depth between individuals with and without bruxism using panoramic radiographs and reported a statistically significant difference between these individuals. However, inspection of their data suggests that this finding was prominent due to the male subgroup, since the mean notch depth appeared to be similar between female bruxers and non-bruxers. To the best of our knowledge, the present study is the first to evaluate both skeletal and muscular parameters in this context concurrently. Despite this comprehensive approach, we found no significant correlation between the AGN size and any of the three masticatory muscles assessed, suggesting that AGN morphology is not directly associated with masticatory muscle function.

Regarding sex-related dimorphic differences, Schutz et al.^24^ reported an increase in AGN depth during pubertal growth in males and proposed that this may be attributed to greater bone apposition at the gonial region during this developmental period. Since the current study is cross-sectional in design and was conducted on an adult population, it does not allow conclusions about the developmental trajectory of the AGN or its changes throughout growth and development. Future studies employing longitudinal designs that track individuals from early childhood through adolescence would be valuable in determining whether the observed sex-related differences in AGN morphology are present early in development or emerge progressively during the pubertal growth phase.

Considering the reported sexual dimorphism, we assessed AGN in different facial types separately for males and females. When comparing males and females from various facial types, an interesting finding emerged: after correcting for mandibular size, the only significant difference between males and females was found in the AFT group. This suggests that males and females with extreme facial types (LFT, SFT) exhibit similar AGN metric characteristics and that the sexual dimorphism of this anatomical feature is more pronounced in individuals with an average facial type. Similar to our results, Mangla et al.^5^ reported no sexual dimorphism in AGN depth when analyzing its size across hyper-, hypo-, and normo-divergent groups.

Interestingly, when comparing AGN across individuals from the three facial types, we observed a significant difference in the presence of AGN in females. More than half of SFT individuals were found with absent AGN, whereas in the LFT group AGN was absent only in 20%. Once AGN was detected as present the extent of its size did not differ between the facial types, in both absolute and corrected measures. These findings imply that the presence of AGN is linked to a vector of facial growth; however, the extent of its size seems not to be related to the facial type.

It is important to highlight that our findings are consistent across both direct and indirect evidence. Specifically, we did not observe a significant association between the AGN size and the masticatory muscle size, nor did we find differences in AGN size across facial types. This consistency strengthens the reliability of our results. If AGN size were indeed related to masticatory muscle strength, one would also expect to observe differences in AGN morphology between individuals with LFT, who are known to have a relatively weak mandibular musculature, and those individuals with SFT, who typically exhibit stronger masticatory muscles^28^. The absence of such differences across both muscle-related and skeletal variables supports the conclusion that AGN size may not be determined by masticatory function.

In searching for possible associations between AGN and other craniofacial and mandibular parameters, we found only a few weak correlations. Interestingly, we found a negative association between AGN and the facial angle, namely, that AGN tends to be greater in more retruded mandibles. This finding is similar to findings by Kolodziej et al.^6^, who also noted a weak negative correlation between the AGN size and the horizontal jaw growth. Although AGN was found to be greater in retruded mandibles, no association was found between the AGN size and the mandibular length. However, this finding contradicts the previous conclusion by Singer et al.^4^ and Salem et al.^3^ that “deep” AGN cases are characterized by a shorter corpus and that these individuals experience less mandibular growth. It is worth noting that these authors analyzed their data on a combined sample of males and females, which could explain such differences. Additionally, we found no associations between AGN and the ramus length and the gonial angle, implying that the AGN size is probably not related to impaired or decreased mandibular growth ability in the normal population. Only a weak negative correlation was detected between AGN and the condyle size, implying greater AGN in cases with smaller condylar sizes. The morphology of the AGN was considered pathognomonic in certain congenital and acquired disorders of the mandibular condyles^29,30^. Patients with unilateral condylar hypoplasia were diagnosed with pronounced AGN on the affected side only^29^. Pathological conditions were beyond the scope of this study; however, it could be presented as a separate topic for future investigation, which may also shed light on AGN development.

It is noteworthy that the AGN size (absolute measures) is associated with all chin parameters, but it is not associated with any of the symphysis measurements. This finding is interesting, since the chin is anatomically a part of symphysis. However, the chin is a bony structure that developed during evolution, and it characterizes uniquely modern *Homo sapiens*, as opposed to the symphysis, which is found in all species^31^. The reason for the presence of a chin in humans is an important open research question that remains to be fully answered. Similar to the morphological characteristics of the chin, our findings suggest that the AGN is a sexually dimorphic feature of the mandible, apparently unrelated to functional factors.

Based on our findings, we hypothesize that the presence of the AGN and the extent of its size are likely governed by distinct factors. The size of the AGN appears to be primarily associated with sex, suggesting a potential role for hormonal influences during pubertal growth or underlying genetic determinants related to sexual dimorphism. These differences may reflect evolutionary mechanisms such as sexual selection, which shape mandibular morphology across populations. In contrast, the presence of an AGN may be linked to the direction of facial growth, which can be genetically driven or influenced by environmental factors such as respiratory patterns and oral habits during developmental periods. In such cases, the presence of the AGN may reflect its role as a fulcrum point, where the mandibular body undergoes rotational changes while the ramus and masticatory muscle attachments remain relatively stable.

Our observations contribute to a broader understanding of mandibular anatomy and its variations, with potential implications across anthropological, anatomical, and clinical domains.

### Study limitations

The current study is cross-sectional in design, which limits the ability to draw conclusions regarding the aging of the AGN. Furthermore, the sample consists exclusively of adults, preventing an assessment of AGN growth and development as well as an investigation of its associations with other craniofacial parameters during the developmental stages.

The prevalence of different facial types in our sample reflected their distribution in the general adult population, resulting in relatively small numbers of individuals with short and long facial types. Therefore, generalizing the results requires further study involving more balanced samples across different facial types.

The peak force of the masticatory muscles was estimated based on the assessment of cross-sectional area size using CT scans; however, functional parameters such as bite force or electromyographic activity were not evaluated in the current study. To better clarify the relationship between AGN size and muscle function, future studies should include these functional assessments.

## Conclusions

The current study presents important findings regarding the considerable AGN variability and its strong sexual dimorphism, as assessed using CT data. Our primary results confirm that approximately one-third of adults do not manifest the AGN. Notably, the presence, but not the size, of the AGN is associated with the direction of facial growth. Individuals with a short facial type showed the highest prevalence of an absent AGN, whereas those individuals with a long facial type exhibited the lowest prevalence. Only a weak correlation was observed between the AGN area and the mandibular plane angle in both sexes. No evidence was found to support an association between the AGN size and reduced mandibular dimensions or a functional relationship in the normal adult population.

## Electronic supplementary material

Below is the link to the electronic supplementary material.

Supplementary Material 1

## Data Availability

The datasets generated during and/or analyzed during the current study are available from the corresponding author on reasonable request.
