# Supplementary material for: Association of antegonial notch size with craniofacial morphology and masticatory muscle dimensions
Source: Sci Rep. 2025 Jul 2;15:23557. doi: 10.1038/s41598-025-08800-x (PMC12223038; doi:10.1038/s41598-025-08800-x)
Supplement: Supplementary file 1 — Supplementary Material 1 [file 41598_2025_8800_MOESM1_ESM.pdf]

Association of Antegonial Notch Size with Craniofacial Morphology and Masticatory Muscle Dimensions

Tatiana Sella Tunis, Dana Rachmiel, Yoav Shapinko, Evgeny Weinberg, Waseem Abboud, and Israel Hershkovitz

Supplementary Table S1. Measurements of the mandible.

Measurement	Definition
Chin thickness	The perpendicular distance between the Pogonion and the line connecting the Menton and B point (the midsagittal aspect) [16].
Chin area	The portion of the symphysis area that is located anterior to the line connecting Menton and B point [16].
Chin width	The distance between the right and left mental tubercles (the frontal aspect) [16].
Symphysis thickness	The distance between the Pogonion and the posterior-most point on the symphysis [16].
Symphysis height	The distance between the superior-most point on the alveolar bone and Menton [16].
Symphysis area	The cross-sectional area of the symphysis in the midsagittal plane [16].
Ramus width	The smallest width of the ramus measured perpendicular to the ramus length [17].
Ramus length	The distance from the highest point on the mandibular condyle to the Gonion [17].
Mandibular body length	The horizontal distance between the anterior margin of the mandible (chin) to a line placed along the posterior border of the ramus [17].
Mandibular body height ratio	The change in the mandibular body height antero-posterior. It is calculated as the ratio of the anterior body height (between premolars) to the posterior body height (distal to the second molar).
Mandibular plane angle	The angle between the MP line and the FH plane [15].
Gonial width	The distance between the Gonion and the deepest point on the concavity that connects the anterior border of the ramus to the mandibular body [14].
Bigonial breadth	The distance between the right and left Gonion.
Gonial angle	The angle formed by the inferior border of the mandibular body and the posterior border of the ramus [17].
Coronoid process height	The vertical distance between the most superior point on the coronoid process and the coronoid process width [14].
Coronoid process width	The distance between the deepest point on the mandibular notch and the anterior border of the coronoid process [14].
Condylar width	The distance between the most lateral and medial points on the condyle head (the axial section parallel to the FH through the middle of the head).
Bicondylar breadth	The distance between the most lateral points of the right and left condylar heads.

Supplementary Table S2. Craniofacial measurements.

Measurement	Definition
Cranial length	The distance between the anterior-most and posterior-most landmarks on the cranial vault (the superior view, the skull is postured parallel to FH).
Cranial breadth	The distance between the most lateral landmarks on the cranial vault measured perpendicular to the cranial length.
Cranial base angle	The angle created between the Nasion, Sella, and Basion (N-S-Ba).
Facial breadth	The distance between the most lateral landmarks on the right and left zygomatic arches.
Anterior facial height	The distance between Nasion and Menton measured perpendicular to FH.
Facial angle	The angle created between the facial (Nasion-Pogonion) and the FH planes [15].

Supplementary Figure S3. Measurements taken on the skull and mandible. Chin and symphysis areas are denoted in red.

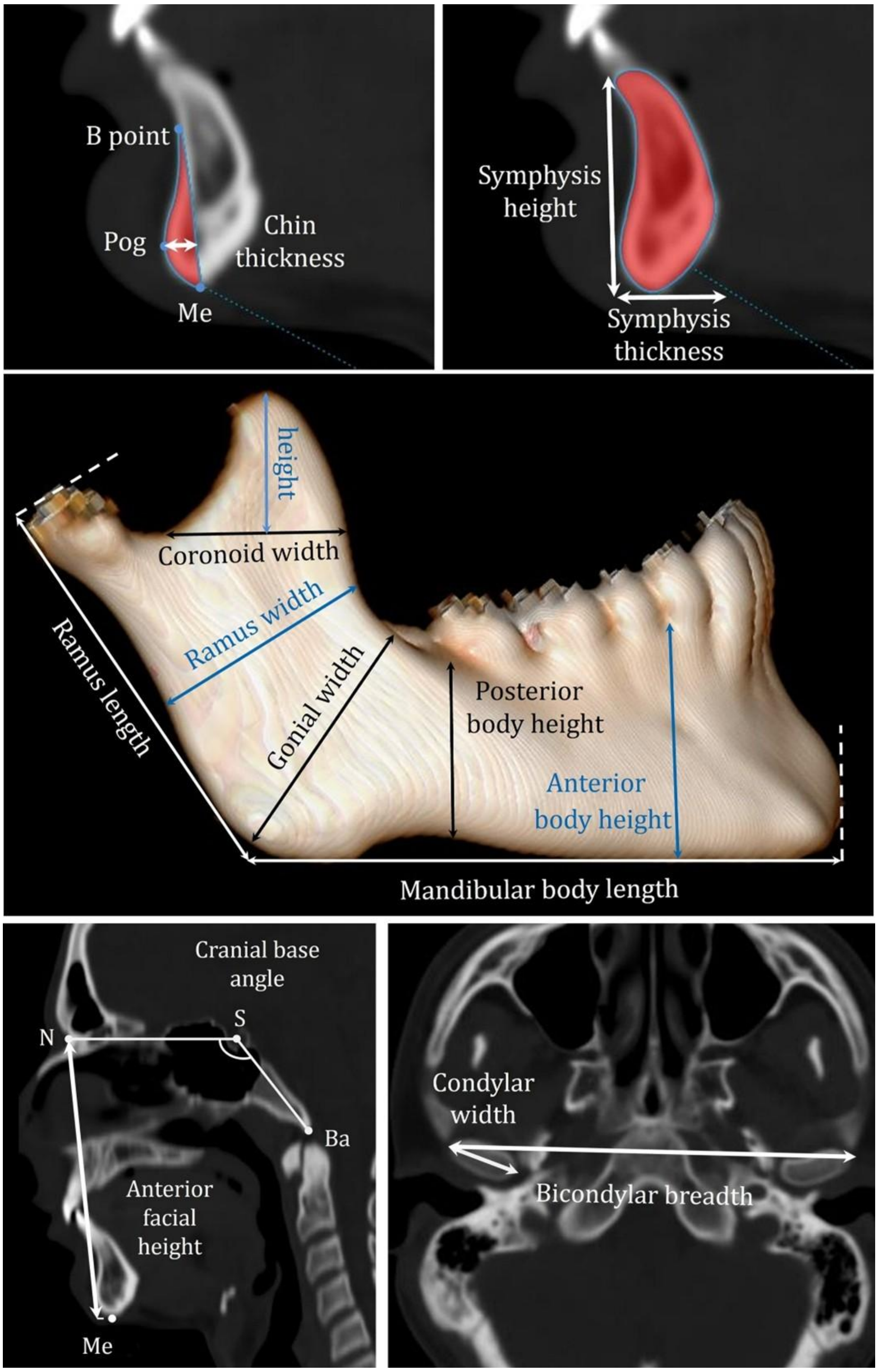

Supplementary Figure S4. Measurements of the masticatory muscle area [18]. The left illustration schematically represents the location of sections 1 (Temporalis m.) and 2 (Masseter m). Section 1 (upper right image) presents the measuring method for the temporalis muscle (the red area). Section 2 (the lower right image) presents the measuring method for the masseter (the red area) and the medial pterygoid muscles (the light gray area encircled by a blue line).

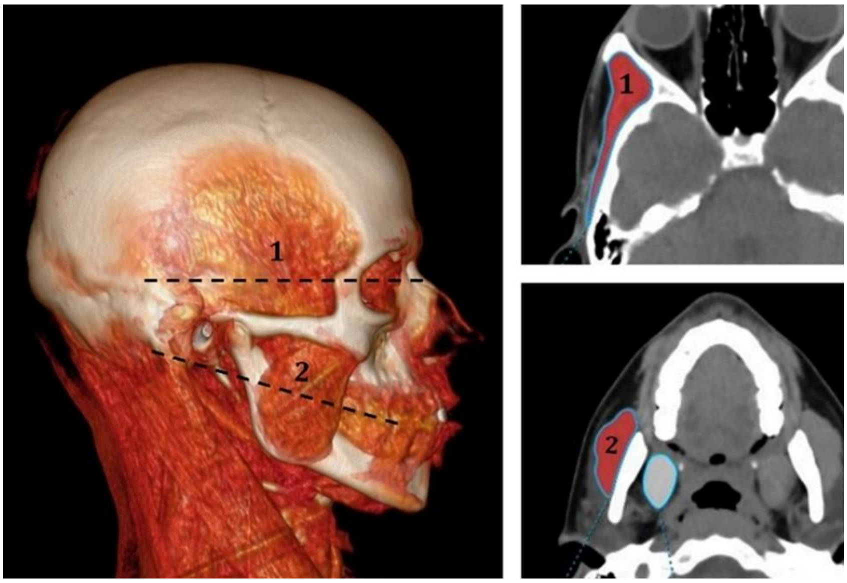

Supplementary Table S5. Correlation coefficients between the AGN area and the muscular parameters in males and females.

Measurement	Sex	Absolute measures			Corrected measures		
		r	*p*-value	$p^{M\ vs\ F}$	r	*p*-value	$p^{M\ vs\ F}$
Masseter CSA	Male	-0.01	0.890	0.794	0.04	0.662	0.380
	Female	0.02	0.772		0.14	0.104	
Medial Pterygoid CSA	Male	0.02	0.755	0.600	0.08	0.308	0.375
	Female	0.08	0.313		**0.18**	**0.035**	
Temporalis CSA	Male	0.00	0.953	0.727	0.05	0.565	0.379
	Female	0.04	0.611		0.15	0.068	

CSA: Cross-sectional area
$p^{M\ vs\ F}$ represent statistical differences in the correlation analysis between sexes.
*Statistically significant results (p<0.05) are denoted in bold.
